# Direct Imaging of Hippocampal Epileptiform Calcium Motifs Following Kainic Acid Administration in Freely Behaving Mice

**DOI:** 10.3389/fnins.2016.00053

**Published:** 2016-02-29

**Authors:** Tamara K. Berdyyeva, E. Paxon Frady, Jonathan J. Nassi, Leah Aluisio, Yauheniya Cherkas, Stephani Otte, Ryan M. Wyatt, Christine Dugovic, Kunal K. Ghosh, Mark J. Schnitzer, Timothy Lovenberg, Pascal Bonaventure

**Affiliations:** ^1^Janssen Research & Development, LLCSan Diego, CA, USA; ^2^InscopixPalo Alto, CA, USA; ^3^Redwood Center for Theoretical Neuroscience, University of California, BerkeleyBerkeley, CA, USA

**Keywords:** calcium imaging, kainic acid, seizure, freely behaving mice, electroencephalography

## Abstract

Prolonged exposure to abnormally high calcium concentrations is thought to be a core mechanism underlying hippocampal damage in epileptic patients; however, no prior study has characterized calcium activity during seizures in the live, intact hippocampus. We have directly investigated this possibility by combining whole-brain electroencephalographic (EEG) measurements with microendoscopic calcium imaging of pyramidal cells in the CA1 hippocampal region of freely behaving mice treated with the pro-convulsant kainic acid (KA). We observed that KA administration led to systematic patterns of epileptiform calcium activity: a series of large-scale, intensifying flashes of increased calcium fluorescence concurrent with a cluster of low-amplitude EEG waveforms. This was accompanied by a steady increase in cellular calcium levels (>5 fold increase relative to the baseline), followed by an intense spreading calcium wave characterized by a 218% increase in global mean intensity of calcium fluorescence (*n* = 8, range [114–349%], *p* < 10^−4^; *t*-test). The wave had no consistent EEG phenotype and occurred before the onset of motor convulsions. Similar changes in calcium activity were also observed in animals treated with 2 different proconvulsant agents, N-methyl-D-aspartate (NMDA) and pentylenetetrazol (PTZ), suggesting the measured changes in calcium dynamics are a signature of seizure activity rather than a KA-specific pathology. Additionally, despite reducing the behavioral severity of KA-induced seizures, the anticonvulsant drug valproate (VA, 300 mg/kg) did not modify the observed abnormalities in calcium dynamics. These results confirm the presence of pathological calcium activity preceding convulsive motor seizures and support calcium as a candidate signaling molecule in a pathway connecting seizures to subsequent cellular damage. Integrating *in vivo* calcium imaging with traditional assessment of seizures could potentially increase translatability of pharmacological intervention, leading to novel drug screening paradigms and therapeutics designed to target and abolish abnormal patterns of both electrical and calcium excitation.

## Introduction

The lives of patients with seizures are impaired not only during convulsive episodes, but also by compromised mood and cognitive function (García-Morales et al., 2008; Mula and Monaco, 2011; reviewed in Lin et al., 2012). One of the main reasons for functional cognitive decline in seizure patients is damage to the hippocampus following seizures; however, the reason for seizure-induced hippocampal damage remains elusive (reviewed by Thom, 2014). Dysregulation of cellular calcium homeostasis has long been implicated as a key component of seizure-related brain damage because of the well-established role of calcium in excitotoxicity (Berdichevsky et al., 1983; Choi, 1988). Since seizure activity has been historically associated with excessive neuronal excitability and synchronization (Jasper and Penfield, 1954), excessive calcium influx through voltage-dependent calcium channels (reviewed by Mehta et al., 2013) during seizures may seem like the most likely cause. However, it is now clear that if seizures are observed in live animals (Matsumoto and Ajmone-Marsan, 1964) or human patients (Truccolo et al., 2011), the observed synchronization does not necessarily involve neuronal hyperactivity. Furthermore, desynchronization is often observed during early stages of seizures while synchronous states may actually contribute to seizure termination (reviewed by Jiruska et al., 2013). Thus, the relationship between seizure activity in the brain and potential increases in intracellular calcium remains unclear. To establish pathologically elevated calcium as a mechanistic link between seizure activity and hippocampal damage, it is necessary to directly observe calcium dynamics in the hippocampus of live animals undergoing seizures; however, due to prior technical limitations, these studies have not yet been performed.

In this study, we combined well established assessments of seizure activity such as behavioral monitoring and whole-brain EEG with functional large-scale real-time microendoscopic imaging of the calcium indicator GCaMP6f in the CA1 region of the hippocampus (Ghosh et al., 2011) in mice treated with a single systemic dose of the pro-convulsant KA. KA induces seizures by direct activation of kainate receptors, which leads to increased excitability in limbic structures (reviewed by Vincent and Mulle, 2009; Carta et al., 2014) and is a widely accepted model of human temporal lobe epilepsy and treatment-resistant epilepsy (Tse et al., 2014; reviewed by Lévesque et al., 2016). Systemic KA injection has the advantage of better experimental control of seizure timing and severity, as compared with genetic animal models of epilepsy or electrical/chemical kindling models that give rise to spontaneous recurrent seizures. Additionally, systemic dosing avoids any unwanted physical effects stemming from direct injection into the brain. To rule out the possibility that the changes in calcium activity following KA administration were associated with excitotoxic effects specific to KA, we imaged animals dosed with two other mechanistically distinct pro-convulsants: PTZ and NMDA.

The integrated approach of physiological calcium imaging used in this study revealed distinct aberrations in hippocampal calcium dynamics that occurred not only during seizures, but also before behavioral or electroencephalographic signatures of convulsive motor seizures became apparent. These included altered intracellular calcium levels that were considerably higher than normal, supporting the idea of excitotoxic levels of cellular calcium as a mediator of neurodegeneration during seizures. Surprisingly, the commonly prescribed anticonvulsant valproate (VA) (Kanner and Balabanov, 2002) did not modify the observed seizure-related abnormalities in calcium dynamics, despite reducing the behavioral severity of KA-induced seizures.

## Materials and methods

### Drugs

The VA, PTZ, NMDA (Sigma-Aldrich, St. Louis MO, USA) and KA (Tocris, Bristol, UK) were formulated before each imaging session in 0.9% sodium chloride (Baxter, Deerfield IL, USA).

### Seizure assessment

Animals were continuously monitored and scored at 2 min epochs according to the most severe stage of a modified Racine scale (Racine, 1972): **0**: no changes in the behavior or EEG; **1**: sudden behavioral arrest and motionless staring, simple low amplitude EEG spikes; **2**: intermittent ear twitches or hiccups, spikes and sharp EEG waves with the amplitudes lower than a typical amplitude observed during the slow-wave sleep; **3**: clonus of the head or forelimbs, tail rigidity, intermediate EEG spikes or poly-spikes with the amplitudes exceeding typical amplitude during the slow-wave sleep; **4**: generalized clonic seizures with rearing; complex high frequency and amplitude EEG spike-waves; **5**: generalized tonic-clonic seizures with loss of posture, falling and/or wild jumping. A seizure stage 3 or higher was classified as a convulsive motor seizure (CMS; Tse et al., 2014; Puttachary et al., 2015). Seizure latency was defined as the time period (in minutes) required to reach CMS. The dose of KA used in this study (15 mg/kg, subcutaneous) evoked seizure symptoms in all animals. Most reached CMS, but none developed stage 5 seizures.

### Animals

**Figure 1:**
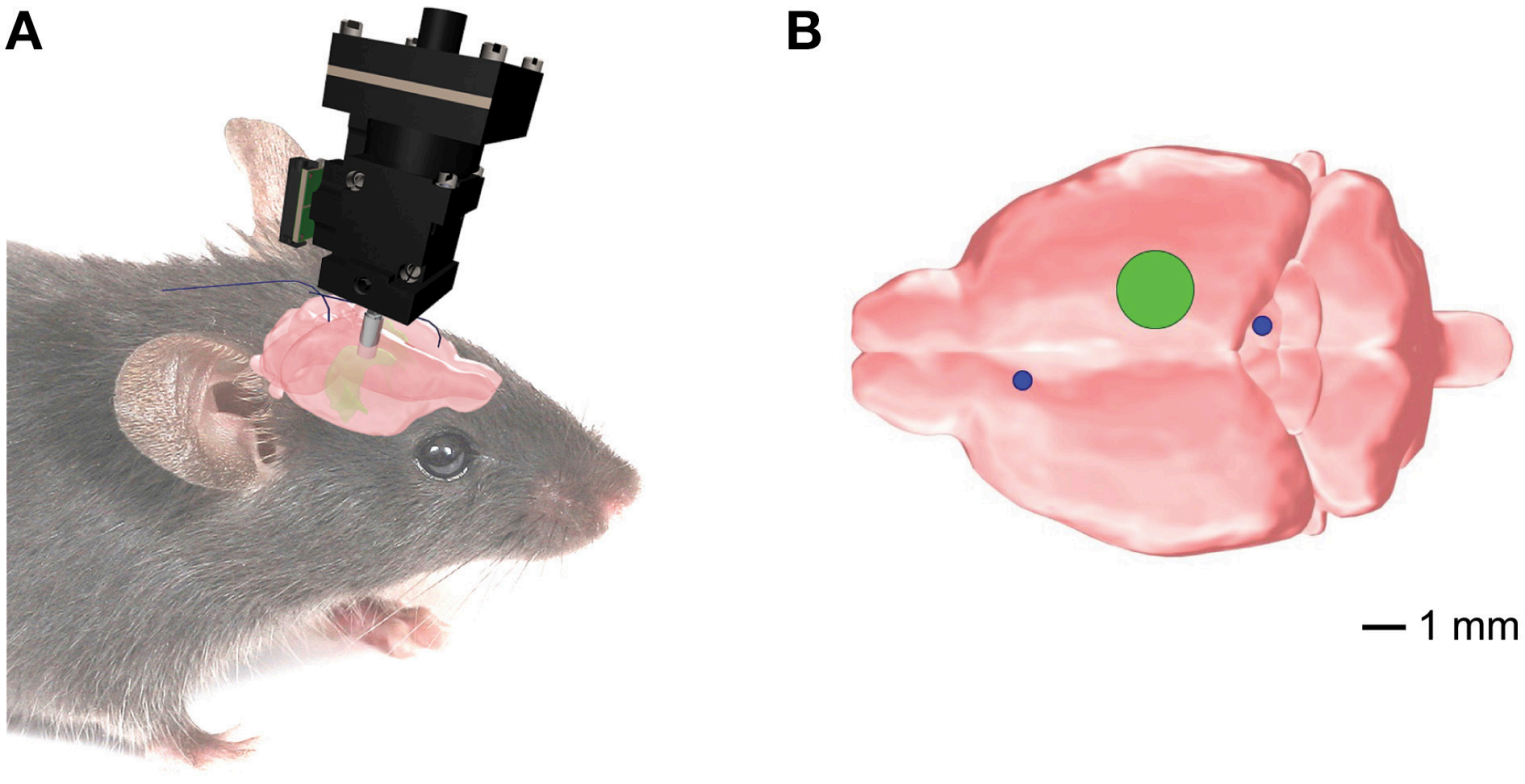
**Position of the implanted EEG electrodes and the microendoscopic calcium imaging device for the combined telemetric and calcium imaging recordings. (A)** Schematic representation of the imaging device and the EEG electrodes. **(B)** Optical guide tube (green circle) stereotaxically centered over AP = −2.3*mm*, ML = 1.89 mm, V = −1.6*mm* from Bregma. The EEG elecrodes (blue circles) for the whole-brain EEG were implanted in the frontal cortex and superior/inferior colliculus, respectively.

All procedures were performed in accordance with the Guide for the Care and Use of Laboratory Animals (US National Institutes of Health). Male C56BL/6J mice (*n* = 22) aged 8–12 weeks underwent two separate surgeries under Isoflurane (1.5–2.5%) with analgesic treatment (Buprenex, 0.05 mg/kg subcutaneous): (1) to inject viral vector (pENN.AAV.CamKII.GCaMP6f.WPRE.SV40, titer 7.3e12 GC/ml, 400–650 nL, diluted in PBS 1:10–1:15) and (2) to implant an optical guide tube over CA1. During the second surgical procedure, a subset of animals also received a subcutaneously implanted telemetric device (PhysioTel F20-EET; Data Sciences International, St. Paul, MN) for polysomnographic recordings (Shelton et al., 2009; Berdyyeva et al., 2014). In the animals equipped for telemetry, we coupled the devices to two sets of stainless steel electrodes: one implanted in the frontal cortex and superior/inferior colliculus for the whole-brain EEG (Figure 1); the second in dorsal nuchal muscles for the electromyogram (EMG; data not shown). The placement of the electrodes for the whole-brain EEG recordings was dictated by our goals to (a) avoid any damage to the hippocampus that would compromise the imaging procedure; and (b) simultaneously examine the whole-brain EEG activity to bring our experimental design closer to the readout in seizure patients. A group of animals with telemetric devices only (*n* = 6) was used as a control to verify that manipulations related to the imaging procedures did not change the seizures' parameters. The mice were allowed to recover for 4–6 weeks. Histological examination was performed after imaging experiments. Some animals (*n* = 7) were excluded from the analysis due to the suboptimal quality of the tissue, imaging artifacts, or low cellular yield.

### Telemetric recordings and analysis

EEG, EMG and locomotive signals were continuously recorded (100 Hz sampling rate, Dataquest A.R.T. software) during the imaging session and scored using Neuroscore software (Data Science International). The EEG recordings were binned into 10-s intervals and classified into epileptiform spike types 0–5 as described in the literature (Tse et al., 2014) and in the “Seizure assessment” section above. The alignment between the imaging system, EEG, and locomotive signals was done by recording, on a separate channel of the polysomnographic system, the state of the imaging system transmitted through an analog channel in nVista and subsequently digitized at 100 Hz (Berdyyeva et al., 2014). Only the animals with < 50 ms discrepancy (*n* = 5) were used to quantify the correlation between the EEG and imaging data.

### Calcium imaging procedure

All imaging sessions were conducted in mice freely behaving in their home cage. At the session onset, an integrated miniature microscope (nVista, Inscopix, Palo Alto) was attached to a skull-mounted baseplate under brief anesthesia (< 1 min, 0.5–1% Isoflurane). Approximately 60 min later the animals were dosed with vehicle. To avoid potential photobleaching, continuous imaging periods lasted less than 10 min, with at least 5 min until the next imaging interval. After collecting 20–30 min of post-vehicle data, the animals were treated with either KA, NMDA (75 mg/kg, intraperitoneal) or PTZ (45 mg/kg subcutaneous) and imaged as above. One group of animals was pre-treated with VA (300 mg/kg intraperitoneal) 15 min before the KA injection. The imaging session was terminated once a seizure exceeding stage 3 was observed (with one exception to observe additional events) or at a maximum duration of 90 min post-KA. At the end of the session, the animals were humanely euthanized.

### Analysis of imaging data

The mean intensity of each video frame was extracted and analyzed using custom written ImageJ and Matlab scripts. The data were z-transformed to determine the wave peak and half-max width (WHMW).

The analysis of cellular effects was performed using PCA-ICA matrix decomposition (Mukamel et al., 2009; Frady and Kristan, 2015) on the following data fragments: post-vehicle (ten 1-min fragments), post-KA but before the first identifiable wave (five 1-min fragments), and during the rapid-flashing and fluorescence build-up (~30 s). PCA-ICA uses correlations in the pixels to factorize the imaging data, which allows the neurons to be separated from the background; further smoothing of neural responses was used to minimize impact of background on measured calcium responses. Non-neuronal components of PCA-ICA decomposition were manually removed. Calcium events were identified by searching each neuronal calcium trace for local maxima with peak amplitude >1 standard deviation (s.d.) from the median of the trace with a decay time >200 ms. The one-tailed Wilcoxon Signed Rank (WSR) test was used to compare event rates across the conditions.

To understand the calcium build-up during the epileptiform activity, we compared the calcium activity of each neuron during the build-up to its typical calcium transients in the post-vehicle time periods. Since the absolute fluorescence intensities can vary across cells based on levels of calcium indicator expression and light intensity, we computed two normalized metrics to characterize the build-up events for each cell. The Log Peak Ratio was calculated by first finding the peak value of the calcium build-up event for a given cell and then dividing that value by the average of the 5 largest calcium transient peaks observed for that same cell during the post-vehicle period prior to KA administration.

$$\begin{array}{l} Log\;Peak\;Ratio\;\left(n\right)\;=\;Log\;\left(\frac{Peak\;Buildup\;\left(n\right)}{mean\;\left(Peak\;Top\;5\;spikes\;\left(n\right)\right)}\right) \end{array}$$

The resultant normalized values were then plotted and reported for all cells on a base-10 log scale. The Log Integral Ratio was calculated by first finding the integral of the calcium build-up event for a given cell and then dividing that value by the average of the 5 largest calcium transient integrals observed for that same cell during the post-vehicle prior to KA administration.

$$\begin{array}{l} Log\;Integral\;Ratio\;\left(n\right)=Log\;\left(\frac{Integral\;Buildup\;\left(n\right)}{mean\;\left(Integral\;Top\;5\;spikes\;\left(n\right)\right)}\right) \end{array}$$

Again, the resultant normalized values were then plotted and reported for all cells on a base-10 log scale.

## Results

### KA administration led to stereotypical patterns of epileptiform calcium activity

**Figure 2:**
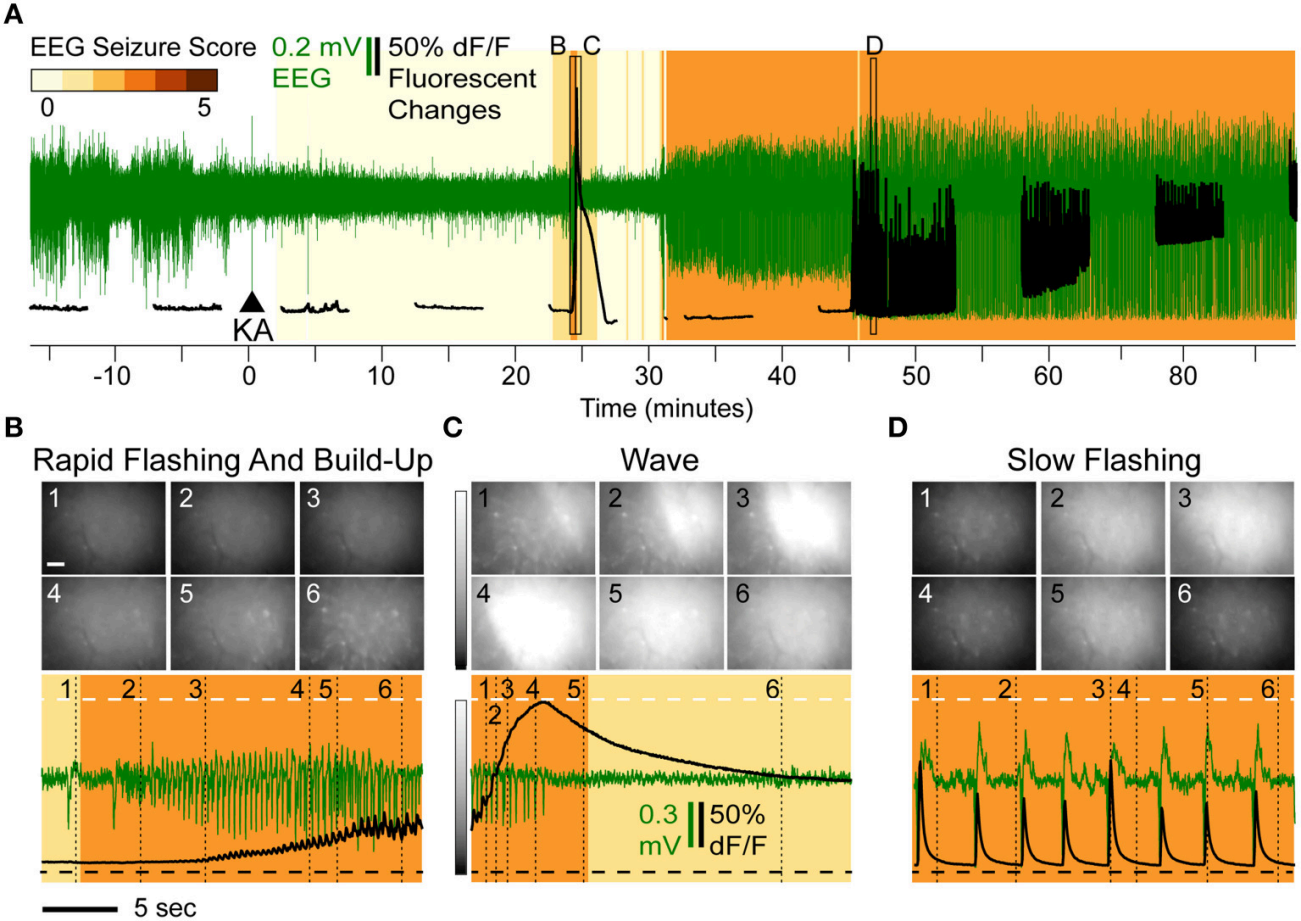
**Overview of Epileptiform Calcium Motifs following KA Administration (15 mg/kg s.c.) in Hippocampal CA1 of a Freely Behaving Mouse. (A)** Simultaneous EEG (green) and calcium (black) activity following KA administration (arrow). The animal in this example never reached a convulsive motor seizure (CMS). Background shading indicates EEG seizure scores. The three stereotypical events are indicated: rapid-flashing and build-up **(B)**, calcium waves **(C)**, and slow-flashing **(D)**. **(B–D)** Expanded views illustrating patterns of rapid flashing and cellular calcium build-up **(B)**, calcium wave **(C)**, and slow flashing **(D)**. The imaging frames (top) show the levels of fluorescence at different time points (Scale: 20 μm, horizontal bar; and 0–200% change in fluorescence, vertical gray scale bar). The mean frame intensity (black) and EEG (green) traces are plotted at the bottom with the imaging frame positions indicated by the numbers 1–6.

The typical progression of events following KA administration is illustrated in Figure 2A. All animals treated with KA (*n* = 8) exhibited a series of flashes in GCaMP6f fluorescence across the entire imaging field that escalated in both frequency and intensity and were accompanied by a steady increase in fluorescence within the cell bodies (Video 1). This pattern (Figure 2B, black trace) was concurrent with a brief cluster of low-amplitude EEG spikes (Figure 2B, green trace) and was consistently and immediately followed by one or two waves of high-intensity fluorescence spreading across the field with a speed of approximately 1 cm/min (Video 1); the fluorescence behind the wavefront dissipated over 1–3 min.

The waves were characterized by a continuous increase in global mean fluorescence intensity followed by a continuous decrease (Figure 2C, bottom, black trace). During the first identifiable wave, the average fluorescence increase was 218% relative to baseline (*n* = 8, range [114–349%]) and was highly significant (*p* < 10^−4^; *t*-test). The average duration of the wave, as assessed as the wave half-max width (WHMW), was 31 s (range [16–69 s], *n* = 8).

On average, the first identifiable wave was observed 19.9 min after KA administration, (range [4.2–44]) and was not associated with any convulsive behaviors. The typical behavioral and EEG (Figure 2C, bottom, green trace) phenotypes were ones associated with stage 1–2 seizures (see Methods, “Seizure Assessment”). In the majority of the animals (6 out of 8), the sequence of rapid flashing, fluorescence build-up and a wave reappeared later in the session but before the onset of motor convulsions (that, if observed during a given session, occurred on average 33 min after the initial wave; range [23–49]).

Half of the animals (*n* = 4) exhibited a third type of epileptiform signature: steady, low frequency (0.2–0.8 Hz) fluorescent flashes (Figure 1D, bottom, black trace) that, in most animals (3 out of 4), also occurred before the onset of the CMS. These varied in intensity but were highly synchronized with epileptiform EEG spikes (Figure 2D, bottom): if a fluorescence spike was detected in a given 100 ms bin (2 imaging frames), the EEG spike was detected within the same bin with significantly greater than chance probability (90.2% on average, [82.5–95%] range, *p* < 0.001, χ^2^ test). In the data combined across animals (*n* = 3), the correlation between detected spike peaks in imaging and EEG was highly significant (*R*^2^ = 0.9994, *p* < 0.0001).

To confirm that the observed abnormalities in calcium activity following KA administration were associated with seizure induction rather than effects specific to KA, we imaged animals dosed with two other mechanistically distinct pro-convulsants: PTZ (*n* = 2) and NMDA (*n* = 2). We observed similar epileptiform fluorescence changes in the majority of animals (50% of animals dosed with PTZ and 100% dosed with NMDA had at least one identifiable wave before the CMS onset; Supplementary Figure 1). The peak fluorescence increases were comparable across groups (KA: average 218%, PTZ animal: 173%, NMDA animals: 209 and 222% increase), however the waves in PTZ and NMDA groups had a faster onset, slower decline and more narrow WHMW (KA: average 31 s, PTZ: 19.9 sec; NMDA: 17.1 and 18.7 s). Interestingly, the wave in PTZ but not NMDA animals was preceded by a series of brief intensifying flashes typically observed with KA.

### Effects of KA administration on individual cells: decreased rate of calcium transients and increased calcium build-up

**Figure 3:**
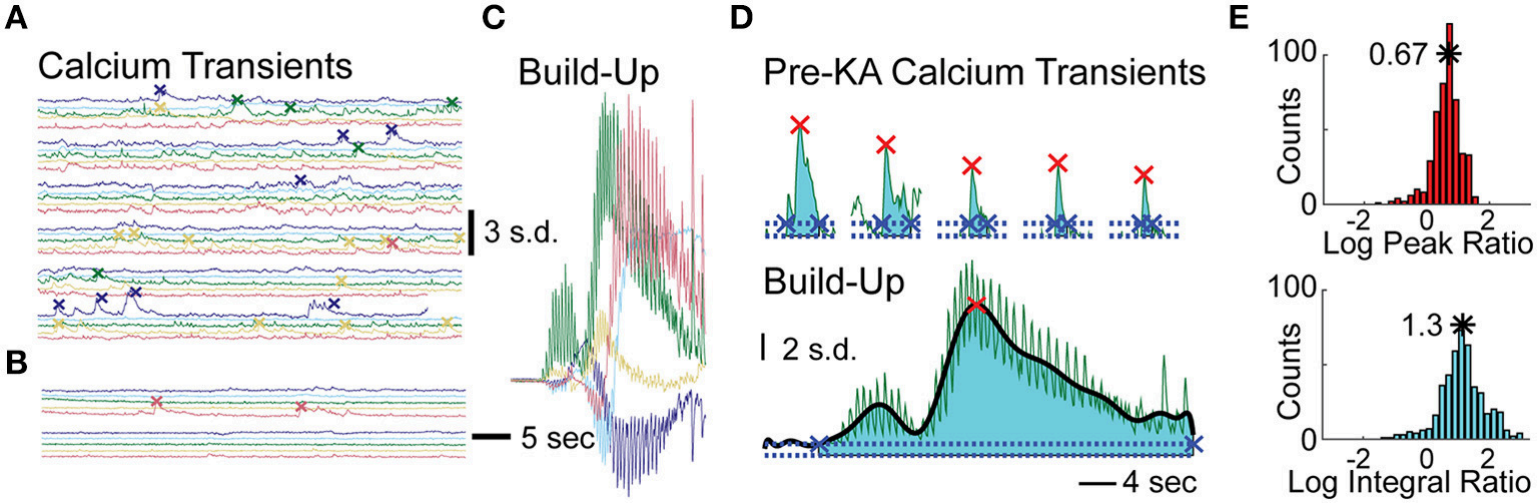
**Analysis of Cellular Events in Hippocampal CA1 of the Mice Treated with KA. (A)** Five representative cellular calcium traces prior to KA administration; detected calcium transient events indicated by “x” marks. **(B)** Same traces after KA administration but before calcium build-up. **(C)** Same traces during the calcium build-up; same scale as in **(A,B)**. **(D)** The peaks (red x) and integrals (cyan shading) of a representative cell: the 5 largest post-vehicle events (top) compared to a typical build-up event (bottom, same scale as on top). The events' beginning and end are marked with blue “x”s. **(E)** Distributions of the build-up event peak amplitude (red histogram, top) and integral (cyan histogram, bottom) combined across animals (_10_log x-axis scale).

To assess effects of KA on cellular calcium activity, we analyzed three periods preceding the calcium wave: post-vehicle spontaneous activity, early post-KA activity, and periods with rapid flashing and build-up. Representative examples of cellular fluorescence intensity changes for these three periods are plotted in Figures 3A–C. Figure 3A illustrates typical post-vehicle calcium transients, Figure 3B shows early post-KA activity; and Figure 3C shows the build-up during the rapid flashing (the same scale was used in Figures 3A–C highlighting the increased intracellular calcium).

To quantitatively examine the cellular effects of KA (100–150 neurons per animal, *n* = 4 animals), we computed differences in the rates of spontaneous calcium transients between post-vehicle and the early post-KA periods. All animals had a significant decrease in event frequency following KA administration (average reduction of 1.5 events/min; *p* < 10^−9^ in each animal, WSR test).

In order to quantify each of the “calcium build-up” events detected in the cellular traces, we computed two metrics and normalized them to the average spontaneous calcium transients observed post-vehicle and prior to KA administration (see Materials and Methods). The Log Peak Ratio is a measure of the maximum fluorescence intensity observed for each cell during the “calcium build-up” event and the Log Integral Ratio is a measure of the fluorescence intensity integrated over the full duration of the “calcium build-up” event (Figure 3D). The rightward shift of the peak and integral ratio distributions (base-10 log scale) computed for each animal and combined across animals (*n* = 4, Figure 3E) indicates higher than normal levels of calcium load in the time periods anticipating convulsive activity (on average, 0.67 ± 0.235 log peak ratio, 5-fold increase in the median peak; and 1.17 ± 0.35 log integral ratio, 20-fold increase in median integral, *p* < 10^−5^ and *p* < 10^−10^ respectively, *t*-test).

### VA did not prevent epileptiform changes in calcium activity following KA administration despite reducing behavioral severity of KA-induced seizures

We investigated whether pre-treatment (15 min) with VA had any impact on post-KA changes in calcium dynamics. The group of animals pre-treated with VA (VAKA, *n* = 11) had significantly reduced severity of behavioral seizures relative to the KA group (KA, *n* = 11) (*p* = 0.0019, one-tailed Mann–Whitney *U*-test) and a lower proportion that reached CMS (27 vs. 91%, *p* = 0.0046, χ^2^ test with Yates' correction; Supplementary Table 1).

**Figure 4:**
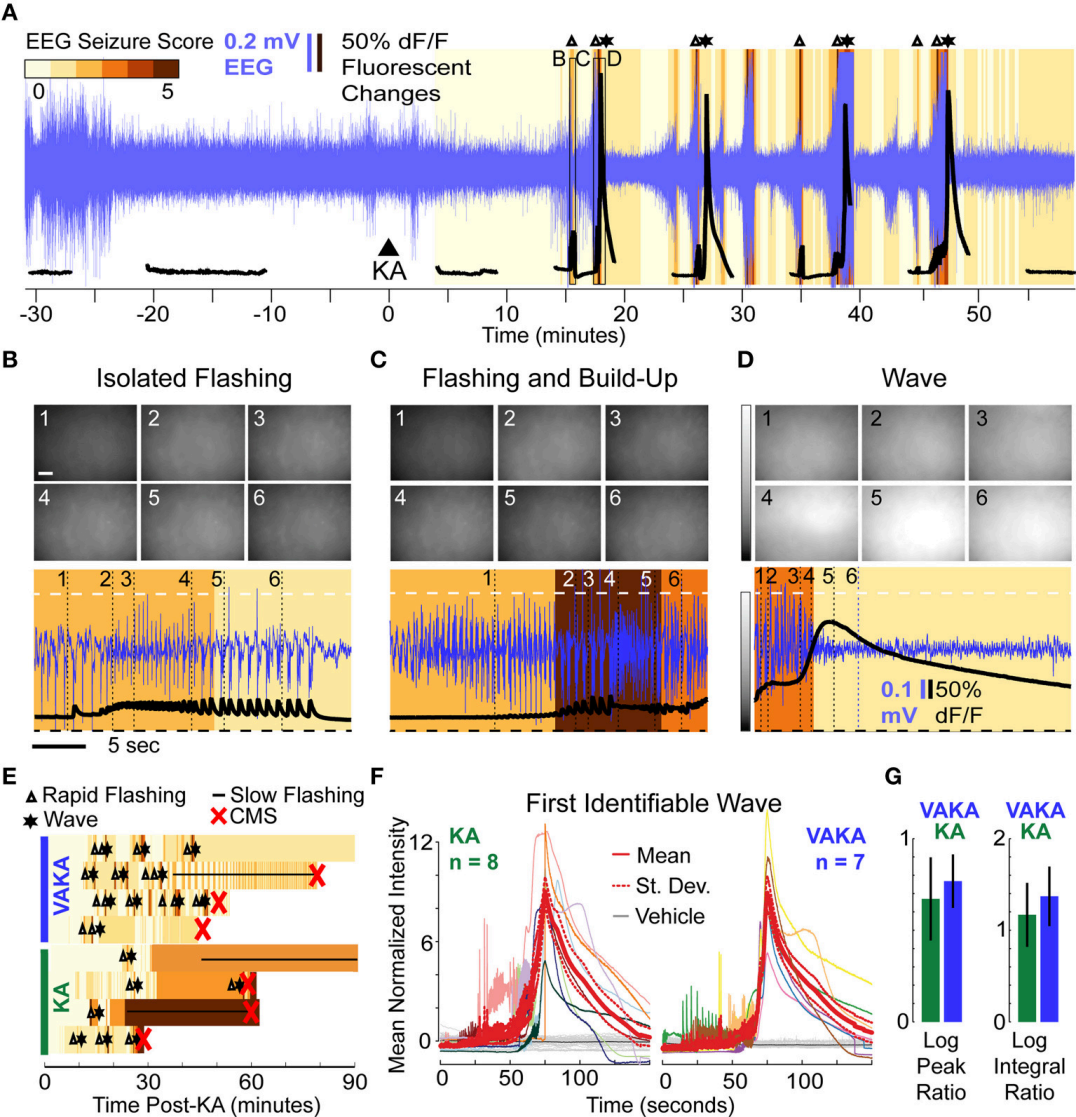
**Epileptiform motifs in CA1 of Mice Pre-Treated with VA (300 mg/kg, i.p.) 15 min Prior to KA Administration. (A–D)** A representative session **(A)** of an animal pre-treated with VA before KA administration (VAKA) exhibited isolated flashing **(B)**, but otherwise had similar epileptifom patterns **(C,D)** as animals treated with KA only. **(E)** Occurrences of stereotypical events (Δ, rapid-flashing and build-up; ^*^, calcium wave; __, slow-flashing) are marked in four representative VAKA (top) and KA (bottom) sessions. Background color indicates EEG seizure scores. Red “x” indicates the onset of CMS. **(F)** Mean normalized fluorescence intensities during the first identifiable wave were aligned with respect to peak time and plotted across time (left: KA animals; right: VAKA animals). **(G)** Analysis of individual neurons with respect to the characteristics of calcium-build-up events (the Log Peak Ratio and Log Integral Ratio) showed no significant differences in the VAKA group compared with the KA group.

A representative imaging session of a VAKA animal is shown in Figure 4A. Pre-treating with VA did not abolish KA-induced epileptiform calcium events (Figures 4B–D). The only noticeable difference observed was a tendency toward bouts of rapid flashes without subsequent calcium waves in the VAKA group (Figure 4B). Similarities in the sequence of epileptiform calcium motifs in representative examples from the KA and VAKA groups are illustrated in Figure 4E.

All imaging videos collected in VAKA animals (*n* = 7) had at least one identifiable calcium wave pattern. Since the VAKA animals reached CMS significantly less often than KA animals, and tended to have longer CMS latencies, the number of waves occurring before CMS was higher than in the KA group (14 waves were detected pre-CMS in 8 KA animals, 19 waves were detected in the 7 VAKA animals). The similar characteristics of the first identified wave in both groups are illustrated in Figure 4F. Statistical comparison of mean wave intensities showed no significant differences between the groups (KA group: 218% increase; VAKA group: 239% increase, *p* = 0.14, *t*-test). Wave duration was similar as well: the WHMW in the VAKA group (23.7 s) was not significantly different from the KA group (31 s, *p* = 0.42; *t*-test).

We performed the same cellular level analysis in VAKA animals as with the KA animals. In VAKA animals, we found a similar reduction in the calcium transient rate in the early post-KA period (average reduction of 1.1 events/min, *p* < 10^−10^ for each animal, WSR); as well as similar responses during the rapid-flashing and calcium build-up motif (Figure 4F, VAKA group: 0.77 ± 0.15 log peak ratio, *p* < 10^−13^, *t*-test; 1.37 ± 0.32 log integral ratio, *p* < 10^−13^, *t*-test, *p* < 10^−9^ WSR; no significant differences between VAKA and KA group: 0.67 and 1.17 for the log and integral respectively).

## Discussion

The decline in mood and cognition in epileptic patients is thought to be a consequence of seizure-induced damage to relevant brain structures such as the hippocampus (Olney et al., 1986; Kienzler et al., 2009; Jafarian et al., 2015). In turn, an established connection between pathologically elevated calcium influx and cellular damage links seizures to neuronal degeneration (Heinemann, 1986; Meldrum, 1986; Staley, 2015; reviewed in Delorenzo et al., 2005; Henshall and Meldrum, 2012). While it is widely hypothesized that abnormal levels of calcium could be a key component of seizure-related excitotoxicity leading to the hippocampal damage (Sztriha et al., 1985; Meldrum, 1986; Nagarkatti et al., 2009; Cao et al., 2015), due to technical limitations, no previous study has addressed whether aberrant calcium dynamics are actually observed in hippocampal neurons of mammals undergoing seizures.

Here, we combined well established assessments of pharmacologically induced seizure activity (i.e., behavioral monitoring and the whole-brain electroencephalography) with large-scale real-time cellular calcium imaging. The use of miniature fluorescence microscopes in freely moving mice suggests that the long-hypothesized pathological patterns of calcium during a seizure in the hippocampus of awake behaving animals not only occurs, but also occurs to a great extent (during the calcium wave, the average fluorescence increase was 218% relative to baseline). In following studies, it would be important to determine if the intense calcium waves that we observed could directly lead to an acute cellular damage in live animals.

An unexpected finding of this study was that the significant deviations from normal calcium dynamics in CA1 arose before (33 min, on average) the onset of motor convulsions. Previous studies utilizing *in-vivo* time-lapse imaging methods (Daniel et al., 2015) compared neuronal morphology before and after seizure induction and found that the most severe seizures acutely damaged dendritic structures (Mizrahi et al., 2004; Rensing et al., 2005), possibly through calcium-dependent actin depolymerization (Zeng et al., 2007). A more recent study found that even brief convulsive seizures (< 5 min) induced dendritic beading and spine loss (Guo et al., 2012) supposedly via the same mechanism, although intracellular calcium was not studied. Our finding that individual hippocampal neurons exhibited a significantly higher than normal calcium load before the onset of motor convulsions indicates that paroxysmal motor convulsions, despite being the most readily noticeable symptom of seizures, could be a mere expression of latent pathological changes within the CNS of seizure patients (reviewed in de Lanerolle et al., 2012; Staley, 2015). Furthermore, the intense calcium waves were observed in the absence of any consistent whole-brain EEG phenotype typically associated with seizure activity. It is also possible that the EEG signals we observed may instead be a result of seizure propagation rather than seizure initiation. Indeed, electrographic definition of the seizure activity could be dependent on the proximity of the electrode placement to the seizure focus. To take into account the whole-brain EEG recordings of our study, we defined the seizure latency as a time required to reach CMS (Tse et al., 2014; Puttachary et al., 2015) that is characterized by clonus of the head or forelimbs, tail rigidity, intermediate EEG spikes or poly-spikes with the amplitudes exceeding typical amplitude during the slow-wave sleep. While we examined the EEG activity on the surface of the cortex to parallel the readout in patients, it is possible that the EEG signatures of seizures would be observed simultaneously with the calcium wave if the electrodes and the calcium imaging device were placed directly in the seizure focus within the hippocampus. These additional studies would be an important subject for future applications of the novel integrated methodology introduced here.

There are two major reasons as to why current seizure medications may be inadequate: (1) incomplete understanding of the underlying pathophysiological mechanisms; (2) inadequate screening methods that favor agents which ameliorate symptoms but not the underlying pathologies. The latter point is illustrated by our finding that VA, a commonly prescribed anticonvulsant (Kanner and Balabanov, 2002), did not normalize abnormal calcium dynamics following administration of KA, despite having protective effect against CMS. We selected VA for this study as it is one of the oldest and most commonly prescribed anticonvulsants in humans (Meunier et al., 1963; Pinder et al., 1977; Tunnicliff, 1999; Who Model List of Essential Medicines, 2013; reviewed by Perucca, 2002). We applied VA at a dose (300 mg/kg) that doesn't change behavior or EEG activity (Roucard et al., 2010) but has protective effect in mice (Brahmane et al., 2010; Löscher et al, 2013); injected systemically with a single dose of KA (Abdel-Rahmanm et al., 1999). We selected the KA seizure induction model as it is one of the most widely used model of seizures that mimics the features and pathophysiology of temporal lobe epilepsy in humans (reviewed by Ben-Ari, 1985; Fisher, 1989; Vincent and Mulle, 2009; Lévesque et al., 2016). In confirmation of the previous studies, we found that VA treatment reduced the severity and delayed the onset of the seizures. However, VA did not alleviate calcium abnormalities observed after KA treatment. While testing the effects of other known anticonvulsants on the epileptifotm calcium motifs remains an important objective of the subsequent studies, the current result demonstrates that VA, a commonly used anticonvulsant, can alleviate motor symptoms but not necessarily the associated pathological processes. This finding can potentially provide an insight as to why current treatments for epilepsy are inadequate (García-Morales et al., 2008; Mula and Monaco, 2011). Therefore, large-scale *in-vivo* calcium imaging could be a valuable screening platform for novel antiepileptic drugs that can abolish the abnormal patterns of both electrical and calcium excitation.

One potential limitation of this study is that is that we only examined one method of seizure induction - acute systemic administration of a chemical proconvulsant agent. While we ruled out a KA-specific pathology by finding similar changes in calcium activity in animals treated with 2 other mechanistically distinct proconvulsants, NMDA and PTZ, the abnormal patterns in calcium activity as described here may not represent de novo epileptiform origins or propagations. NMDA directly activates NMDA receptors leading to the initiation and spreading of a depolarization that is often used to evoke and study infantile spasms (reviewed by Stafstrom and Holmes, 2002; Galanopoulou, 2013), status epilepticus (SE, reviewed by Wasterlain et al., 2013), and seizure-related excitotoxic damage (Cruz et al., 2003; Milhaud et al., 2003; Chang-Mu et al., 2010). In contrast to KA and NMDA, PTZ antagonizes GABAergic inhibition and is often used to model acute generalized convulsions (reviewed by Löscher, 2009). It is also possible that the different modes of seizure induction such as electrical kindling or spontaneous seizures in the genetic animal models that cause lesser damage in the hippocampus evoke different or no epileptiform calcium motifs. These additional studies will be the subject of future applications of the technique. Interestingly, however, a recent study by Cao et al. (2015) demonstrated similar KA-induced patterns of calcium activity in cultured hippocampal neurons. The second limitation of this study is that the changes in intracellular calcium were inferred from changes in fluorescence of the GCaMP6f calcium sensor. The effects observed here deviated from normal activity patterns to such an extent that they could saturate the sensor and therefore compromise its ability to accurately report changes in calcium levels. In addition, the non-continuous imaging regime used to minimize potential phototoxicity and the conservative statistical analysis could potentially result in an underestimation of the magnitude of seizure-related abnormalities in calcium dynamics.

Calcium imaging in freely behaving animals at single cell resolution opens a new possibility for investigating molecular mechanisms of seizure pathophysiology previously inaccessible by traditional methods. Our findings support calcium as a candidate signaling molecule on a pathway connecting seizures to subsequent cellular damage. Given that calcium is one of the most important neuronal second messenger systems (Berridge, 1998), controlling a variety of functions including cell integrity (Zeng et al., 2007), energy metabolism (Zsurka and Kunz, 2015) and cell death (Paschen, 2003), the novel screening paradigm introduced here may lead to a better understanding of the mechanisms behind various CNS diseases and potentially increase translatability of novel pharmacological intervention, leading to novel drug screening paradigms and therapeutics.

## Author contributions

Designed the experiments: TB, LA, and PB. Performed the experiments: TB, LA, and RW. Analyzed data: TB, PF, JN, YC, SO, RW, and CD. Wrote the manuscript: TB, PF, RW, and PB. Contributed tools/materials/expertise: TB, CD, KG, MS, TL, and PB. Supervised: MS, KG, TL, PB.

## Funding

This research was funded by Janssen LLC.

### Conflict of interest statement

TB, LA, YC, RW, CD, TL, and PB are paid employees at Janssen Pharmaceutical Research & Development, LLC; JN, SO, and KG are paid employees at Inscopix; KG is a founder and CEO of Inscopix; and MS is a Chief Scientist at Inscopix. This does not alter the authors' adherence to the journal's policies on sharing data and materials. PB and PF declare that the research was conducted in the absence of any commercial or financial relationships that could be construed as a potential conflict of interest.

## Supplementary material

Video 1 **Overview of Stereotypical Epileptiform Motifs**. Videos of the epileptiform motifs are presented in real-time. Changes in calcium sensor fluorescence are presented on a log-contrast scale in order to visualize the changes within individual cells during the flashing and wave epileptiform patterns, which differed in intensity by orders of magnitude. Concurrent EEG activity is plotted below the imaging video. M190 KA: Rapid Flashing & Build Up: After a period of quiescence, flashes of calcium activity (concurrent with low-amplitude epileptiform EEG waveforms) are visible. As the flashes continue, individual cell bodies gradually increase in fluorescence with a varied timecourse. Calcium Wave: Immediately following the build-up, one or two concurrent waves propagate across the field of view. EEG activity shows no identifiable phenotype. After the wave, the increased fluorescence slowly decays. Slow Flashing: After one or more sequences of rapid flashing and waves, the calcium and EEG activity in some animals enters a prolonged period of slow calcium flashing/EEG spiking. The calcium within cell bodies either remains elevated or follows the flashing pattern. M182 VA+KA: In animals pre-treated with VA, isolated rapid-flashing and build-up with no subsequent calcium wave was observed; otherwise the calcium epileptiform events were similar to the KA group.

Supplementary Figure 1 **Mean normalized fluorescence intensities during the first identifiable wave were aligned with respect to peak time and plotted across time (red and blue, NMDA treatment; green, PTZ treatment)**. Same scale as Figure 4F.

Supplementary Table 1 **Assessment of seizure severity in different groups of mice**. A seizure stage 3 or higher (“Materials and methods, Seizure assessment”) was classified as a convulsive motor seizure (CMS). Seizure latency was defined as the time period (in minutes) required to reach CMS. No significant differences were found between mice implanted with telemetric device only (“TEL”), and mice implanted with both telemetric and imaging device (“TEL+IMG”). Pre-treatment with VA significantly reduced behavioral severity of seizures (*p* = 0.0019, one-tailed Mann–Whitney *U*-test), and significantly lowered the proportion of animals that reached CMS (10 out of 11 animals in “KA” group vs. 3 out of 11 in “VA+KA” group; p = 0.0046, one-tailed chi-square test with Yates' correction).
