# Supplementary material for: Direct Imaging of Hippocampal Epileptiform Calcium Motifs Following Kainic Acid Administration in Freely Behaving Mice
Source: Front Neurosci. 2016 Feb 29;10:53. doi: 10.3389/fnins.2016.00053 (PMC4770289; doi:10.3389/fnins.2016.00053)
Supplement: Supplementary Figure 1 — Mean normalized fluorescence intensities during the first identifiable wave were aligned with respect to peak time and plotted across time (red and blue, NMDA treatment; green, PTZ treatment). Same scale as Figure 4F. [file DataSheet1.docx]

SUPPLEMENTARY TABLE 1

| **Animal ID** | **Surgery** | **Treatment** | **Severity** | **CMS Latency (min)** |
| --- | --- | --- | --- | --- |
| M139 | TEL+IMG | KA | 4 | 49 |
| M145 | TEL+IMG | KA | 3 | 32 |
| M157 | TEL+IMG | KA | 4 | 26 |
| M163 | TEL+IMG | KA | 4 | 76 |
| M165 | TEL+IMG | KA | 4 | 31 |
| M166 | TEL+IMG | KA | 4 | 29 |
| M171 | TEL+IMG | KA | 3 | 28 |
| M186 | TEL+IMG | KA | 3 | 90 |
| M189 | TEL+IMG | KA | 3 | 61 |
| M190 | TEL+IMG | KA | 2 | N/A |
| M191 | TEL+IMG | KA | 3 | 59 |
| M172 | TEL+IMG | VA + KA | 1 | N/A |
| M173 | TEL+IMG | VA + KA | 2 | N/A |
| M174 | TEL+IMG | VA + KA | 1 | N/A |
| M175 | TEL+IMG | VA + KA | 1 | N/A |
| M177 | TEL+IMG | VA + KA | 3 | 43 |
| M179 | TEL+IMG | VA + KA | 1 | N/A |
| M182 | TEL+IMG | VA + KA | 4 | 48 |
| M183 | TEL+IMG | VA + KA | 2 | N/A |
| M184 | TEL+IMG | VA + KA | 2 | N/A |
| M188 | TEL+IMG | VA + KA | 3 | 78 |
| M192 | TEL+IMG | VA + KA | 2 | N/A |
| M201 | TEL | KA | 2 | N/A |
| M202 | TEL | KA | 3 | 63 |
| M203 | TEL | KA | 4 | 35 |
| M204 | TEL | KA | 3 | 69 |
| M205 | TEL | KA | 3 | 26 |
| M206 | TEL | KA | 3 | 73 |

## Supplementary Table 1: Assessment of seizure severity in different groups of mice. A seizure stage 3 or higher (“Materials and methods, Seizure assessment”) was classified as a convulsive motor seizure (CMS). Seizure latency was defined as the time period (in minutes) required to reach CMS. No significant differences were found between mice implanted with telemetric device only (“TEL”), and mice implanted with both telemetric and imaging device (“TEL+IMG”). Pre-treatment with VA significantly reduced behavioral severity of seizures (p = 0.0019, one-tailed Mann–Whitney *U*-test), and significantly lowered the proportion of animals that reached CMS (10 out of 11 animals in “KA” group vs 3 out of 11 in “VA+KA” group; p = 0.0046, one-tailed chi-square test with Yates’ correction).


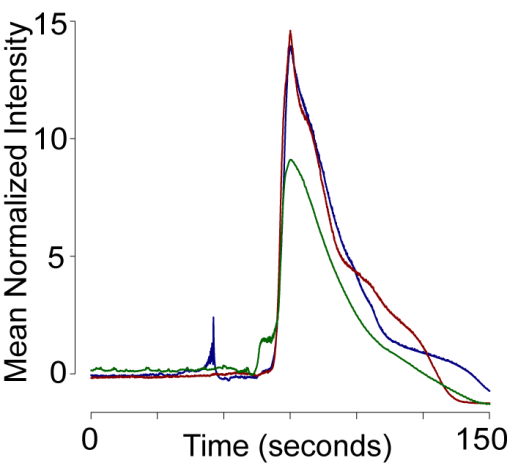


**Supplementary Figure 1**. Mean normalized fluorescence intensities during the first identifiable wave were aligned with respect to peak time and plotted across time (red and blue: NMDA treatment; green: PTZ treatment). Same scale as Figure 4F.
